# Towards improved biomonitoring tools for an intensified sustainable multi‐use environment

**DOI:** 10.1111/1751-7915.12395

**Published:** 2016-07-29

**Authors:** Jan Roelof van der Meer

**Affiliations:** ^1^ Department of Fundamental MicrobiologyUniversity of Lausanne1015LausanneSwitzerland

## Abstract

The increasing use of our environment for multiple contrasting activities (e.g. fisheries, tourism) will have to be accompanied by improved monitoring of environmental quality, to avoid transboundary conflicts and ensure long‐term sustainable intensified usage. Biomonitoring approaches are appropriate for this, since they can integrate biological effects of environmental exposure rather than measure individual compound concentrations. Recent advances in biomonitoring concepts and tools focus on single‐cell assays and purified biological components that can be miniaturized and integrated in automated systems. Despite these advances, we are still very far from being able to deploy bioassays routinely in environmental monitoring, mostly because of lack of experience in interpreting responses and insufficient robustness of the biosensors for their environmental application. Further future challenges include broadening the spectrum of detectable compounds by biosensors, accelerate response times and combining sample pretreatment strategies with bioassays.

## Introduction

With the ever‐expanding human population on our planet, and with increased exploitation of natural resources and concomittant destruction of natural (‘undisturbed’) environments, human societies become pressed for constant and more optimized monitoring of environmental quality (Bakker, 2012; Maxwell *et al*., 2015). More and more, social, agricultural and industrial activities compete for the same environment(s) because of the lack of available alternatives, enforcing the need for their sustainable intensification (Garnett *et al*., 2013). As examples, fisheries and aquaculture farms compete for the same coastal areas as tourism, but both are affected by the effluents and discharges of nearby cities, harbours and factories. Industry's appetite for regular and rare metals entails important mining activities at land or at sea, yet these are creating toxic waste that influences large areas of habitable land, contaminating valuable agricultural areas and impacting natural reserves (Hudson‐Edwards, 2016). Human activities imply making ecosystem tradeoffs at regional and global level; yet, no activity is any longer without immediate direct and indirect effects on others (Goldstein *et al*., 2012; Polasky *et al*., 2014). Hitherto piecemeal developed environmental law and regulations will thus need to be improved to better regulate regional and international environment usage, to curtail transboundary effects and enable multi‐use activities (Kurukulasuriya and Robinson, 2006).

Planning multi‐use activities in regional environmental compartments (that in reality have little boundaries because of fluxes between them) requires setting boundary conditions that enable potentially competing or conflicting activities to be carried out simultaneously. These boundary conditions can involve setting parameters for, for example, quota of fish or animals, species diversity, volumetric water or land use, but in many cases will involve defining environmental quality standards to ensure that resource utilization as a result of one activity is not harming the next. Ecotoxicological and environmental research will help to define the standards and parameters to be focused on (e.g. chemicals, pathogens), but how will such parameters be routinely and intensively measured? Classical measurement approaches, such as taking isolated samples, transporting those to highly specialized laboratories, purifying and analyzing them for the full spectrum of compounds, will likely fail for the task. Despite being the ‘gold’ standard, deep coverage multi‐compound analysis is too expensive and cumbersome for monitoring purposes, and its results are too complicated to interpret. In contrast, a multitude of recent projects, both at national and international levels, try to advance analytical research that could potentially solve one or more of the challenges presented by the increased need for improved monitoring (e.g. European Commission, 2014). Ideally, such new tools and concepts will also lead to decreased measurement complexity and costs, while maintaining high quality information. The main advances presented by current projects lay in the areas (i) of development and improvement of alternative measurement strategies (e.g. biosensors, bioassays), (ii) of alternative sampling techniques [e.g. platforms (Brinkmann and Eisentraeger, 2008), robots and swarms (Koprowski *et al*., 2013; Duarte *et al*., 2016), or passive samplers (Greenberg *et al*., 2014)], and (iii) of measurement accessibility [e.g. Internet access to monitoring networks, public awareness and do‐it‐yourself teams (Landrain *et al*., 2013)].

## Biosensors and biomonitoring

There is a long tradition of using living organisms for environmental quality monitoring (and toxicology more in general). The assumption of using living organisms is that in contrast to chemical compound analysis, they display the integrated direct, chronic and indirect effects of exposures to their living environment (Spurgeon *et al*., 2010). Such integrated measurements could be particularly useful for environmental quality monitoring, since it allows a prediction of probabilities of occurrence of harmful effects on living organisms (including ourselves). Evidently, it is not trivial to measure effects on living organisms, link effects to chemical exposure and project longer term effects. A good option is to examine biomarkers in organisms exposed at the sites of interest, but this can be cumbersome, invasive and time‐intensive (Galloway, 2006; Hook *et al*., 2014). More importantly, observed *in situ* responses ideally need to be compared with organismic responses under calibrated conditions, which is undoable and unethical particularly for higher living organisms (e.g. fish, rats, dogs, etc.) (Scholz *et al*., 2013). Chemical analysis excels at measuring individual compounds to high sensitivity and selectivity at very low concentrations in defined samples. But to project from such measurements to compound bioavailability, organismal exposure and to biological effects is very challenging (Escher and Hermens, 2004; Brack *et al*., 2016). Research in the past 10–20 years has advanced several alternative bioassay types, mostly based on single‐cell microorganisms, cell lines, or purified biological components. These retain the potential to integrate chemical exposures to a reasonable realistic representation of biological effects, but are more simple and easy to standardize, raise less ethical concerns and have the advantage for being deployable at large throughput (Dardenne *et al*., 2007).

**Figure 1:**
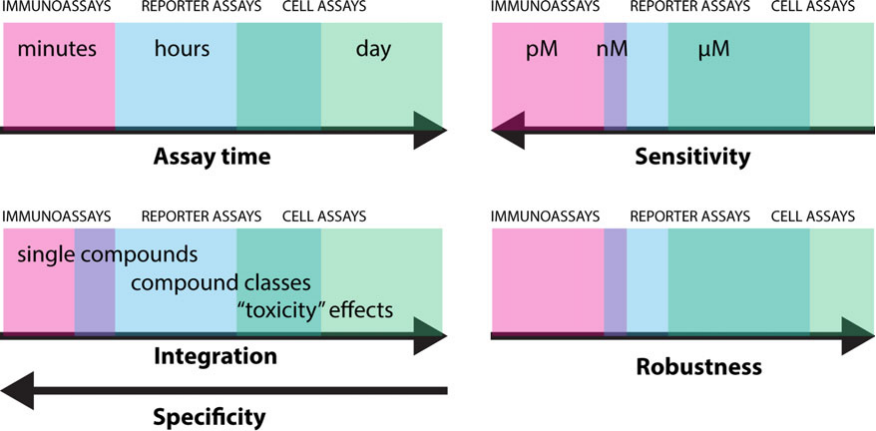
Global performance indices of current bioassays (immunoassays, bioreporters and eukaryotic cell assays), in terms of assay time, sensitivity, measurement type (integration and specificity) and robustness to environmental samples.

Single cell microorganisms, mostly bacteria, have been intensely used for the development of bioassays, covering both detection of single compounds or compound classes, or leading to integrative measures of various cellular effects (e.g. toxicity, DNA damage, oxidative stress) (Belkin, 2003; Yagi, 2007; Woutersen *et al*., 2011). Innate reactions from microbial cells can be used as response marker, such as cellular respiration or bioluminescence, but a strong focus has been placed on the engineering of synthetic circuits and deployment of reporter proteins that are *de novo* produced by the cell in response to target detection (*bioreporters*) (Daunert *et al*., 2000; van der Meer *et al*., 2004). The advantages of using reporter proteins are the signal amplification by the cell as a result of combined transcription–translation and a rather low background signal in the absence of the target (van der Meer *et al*., 2004). The past two decades of research have seen impressive bioreporter engineering efforts, both conceptually and in the variety of reporter designs, yielding a range of detectable compounds and variable outputs (van der Meer, 2010). Different assays using reporter cells have been developed covering a variety of sample types, and with method of detection limits in the order of nM‐μM compound (Fig. 1). Several examples have demonstrated that bioreporter assays are robust and produce quantitative data, which are comparable to remote laboratory‐performed chemical analysis, but have the advantage of requiring little sample preparation and being carried out on‐site practically in real‐time (e.g. Trang *et al*., 2005; Paton *et al*., 2009; Siegfried *et al*., 2012; Brussaard *et al*., 2016). Bioreporters are extremely simple and cheap to produce, quality control is easy and their handling requires little expert knowledge, which is exemplified by growing interest from do‐it‐yourself communities. A continuing disadvantage remains that bioreporters carry synthetic genetic circuits, which for most countries necessitates working under specific biosafety legislation and restricts applications outside qualified laboratories.

## *In vitro* assays

*In vitro* assays, such as immunoassays, can overcome many disadvantages posed by whole cell living bioreporters. Instead of using the cell itself as actuator for response to a target, *in vitro* assays use isolated and purified biomolecules (Lechuga, 2005). These biomolecules can either directly interact with a target molecule and transform the interaction to measurable response (e.g. enzyme reactions), or bind very specifically without further transformation (e.g. antibodies). Both enzyme‐ and immunoassays are widely deployed in a variety of protocols and instruments, mostly but not exclusively, in biomedical areas. In addition to assays with single purified biomolecules, also multiple purified components of the cell can be used ensemble to reconstitute cellular reactions upon target recognition. This was recently successfully demonstrated by creating an *in vitro* transcription–translation system that recreates the bioreporter gene circuit response, but without the disadvantage of deploying genetically modified organisms in the test itself (Pardee *et al*., 2014). The power of *in vitro* assays is their increased detection specificity and selectivity in comparison to bioreporter cells, in particular when the sensor biomolecule can be optimally matched to the target (as antibodies can frequently achieve). Furthermore, they can be optimized to such a degree that the detection sensitivities decrease to the lower nM or pM‐range (Fig. 1) (Estevez *et al*., 2014). On the downside, *in vitro* assays require more handling in assay preparation, reagents can be expensive or difficult to obtain, and the increased assay sensitivity results in lower robustness to environmental samples.

## Eukaryotic cell assays

Finally, several groups have developed assays based on living eukaryotic cells, the reasons being that prokaryotes, despite their simplicity, are fundamentally different than eukaryotes. Therefore, extrapolation from prokaryotic cell responses has limited predictability to eukaryotes. Eukaroytic cell assays range from reporter constructs in, for example, yeast (Bakhrat *et al*., 2011) or rat cell lines (Pieterse *et al*., 2013), use of living algae (Giardi *et al*., 2001) or to purified fish‐cell lines (Brennan *et al*., 2012). Great efforts have been made to develop the most useful and rapid marker assay end‐points, while maintaining a biologically integrative response. Research groups have relied on specific markers (Escher *et al*., 2006), reporter expression (Pieterse *et al*., 2013), or combinations of cellular stains, morphology differences, subcellular localization differences or changes in cell–cell contacts (Brennan *et al*., 2012). In some cases, the cell itself gives an innate useful, easily measurable and sensitive output. For example, the photosystem fluorescence of algae can be measured uninvasively and is a fast and sensitive readout for exposure to herbicides (Giardi *et al*., 2001; Escher *et al*., 2006; Guo and Tan, 2013).

## Automated and self‐deployable bioassays

**Figure 2:**
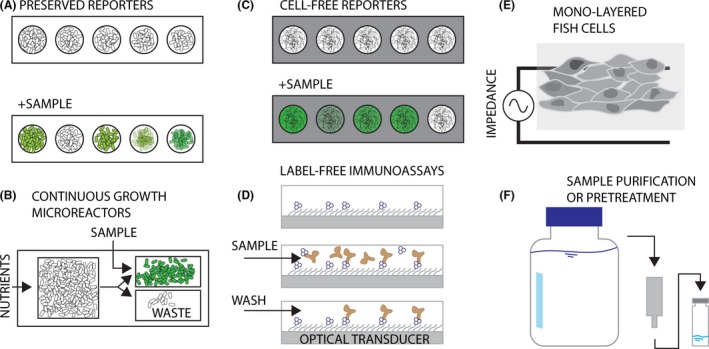
Recent conceptual bioassay advancements. (A) Array‐type bacterial bioreporters with different target specificities but same output signal type (Melamed *et al*., 2012). (B) Continuous‐growth bioreporter chips for semi‐continuous measurements (Buffi *et al*., 2016). (C) Cell‐free reporter systems embedded in, for example, paper (Pardee *et al*., 2014). (D) Miniaturized label‐free immunoassays that can be parallelized for multi‐compound detection (Duval *et al*., 2012). (E) Impedance sensors of tight contacts of, for example, confluent fish gill‐cell layers (Brennan *et al*., 2012). (F) Sample purification or pre‐concentration steps to reduce sample complexity (Brack *et al*., 2016).

**Figure 3:**
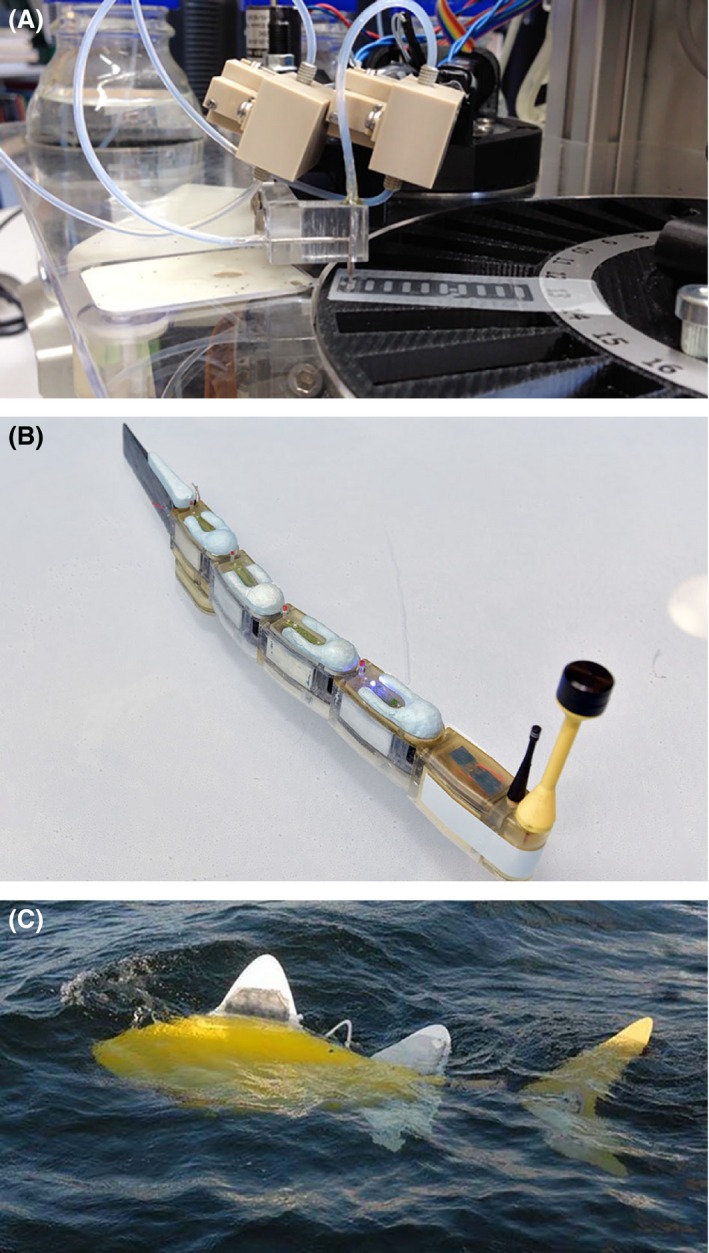
Current ideas on (bio)sensor embodiments for monitoring purposes. (A) *BRAAVOO* automated sampling and readout system for bioluminescence from bacterial bioreporter chips to be embedded on a marine buoy [ref (BIOLUM)]. (B) *Envirobot*, an aquatic robot with multiple sensor segments designed for source tracking and sampling missions [ref (Nano‐Tera)]. (C) *SHOAL*, a marine robofish equipped with physical and chemical sensors that may be deployed in swarms [ref (SHOAL)].

While bioassays have clear advantages in terms of interrogating and integrating biological signals, they do not necessarily make the whole measurement process simpler and interpretation of the cellular responses remains difficult. How can such assays become useful for the types of monitoring that societies might need in future? One aspect is miniaturization. Because of their small size, cells or cell components can potentially be contained in very small devices or instruments with little energy usage that could be deployed on a routine base (van der Meer and Belkin, 2010). For example, arrays of bioreporters can be produced in small format, which consist of multiple bacterial strains with different detection specificity but with the same output (Fig. 2A) (Elad *et al*., 2008, 2011; Melamed *et al*., 2011). This could lead to single‐use cartridges with lyophilized bioreporter strains covering different target specificities, which may be assembled in a flexible manner depending on the expected samples. The reporter cells in the cartridges are activated by the addition of the sample, and may be deployable in dedicated small instruments that can readout the signal and interpret the results (Fig. 3A). The multi‐strain cartridges might be composed of bioreporters targeting single compound classes of particular interest and of those responding to general toxicity of the sample. This would produce a sort of ‘early‐warning’ system combined with specific compound analysis. Alternatively, bioreporter cells may be continuously cultured on dedicated microfluidic platforms (DeBusschere and Kovacs, 2001; Buffi *et al*., 2016) (Fig. 2B) to have constantly active cells that can be exposed at any moment to an aqueous sample, with a specific readout and signal interpretation. Multiple such microfluidic growth‐exposure platforms could be combined to gain capacity of targeted multi‐analyte analysis for longer durations in automated manner.

Recent developments with *in vitro* assays have shown that it is possible to impregnate and preserve a full transcription–translation bioreporter gene circuit on paper, which could lead to extremely easy readouts while maintaining high selectivity and sensitivity (Fig. 2C). This principle could be integrated in small hand‐held devices as well as dedicated instruments that perform automated sample analysis, much in the same way as for living bioreporter cells. Multiple groups advance the miniaturization of label‐free immunoassays, which could retain the advantage of the high selectivity and sensitivity of immunoassays but simplifying and automizing sample and reagent flow (Fig. 2D) (Duval *et al*., 2012). Although many of these efforts are driven by biomedical goals, their developments could also be profitable for environmental applications.

Further advances in miniaturization also led to the development of electrode chips on whose surface confluent cell layers are grown. Such layers are characterized by tight contacts between cells. The extent of tight contacts can be sensitively measured by impedance (Fig. 2E), and is frequently reduced by exposure to contaminated water (Curtis *et al*., 2009; Widder *et al*., 2015). Particularly fish‐cell layers such as from the gill or gut, are very stable and can be maintained without great difficulties for months, which could therefore potentially be deployed in aqueous environments for continuous monitoring of harmful substances (Widder *et al*., 2015). The afore‐mentioned algal photosystem II fluorescence is also a very stable parameter and algae are easy to maintain. Such systems therefore could also be integrated in miniaturized flow‐through cells in automated monitoring stations to guard against sudden toxicity or contamination in the water.

## Buoys, swarms and robots

Miniaturized bioassays might easily be integrated in devices with a different sampling strategy that no longer relies on the single‐sample manual intervention strategy. Similar to physical sensors, also miniaturized biosensors may be integrated in, for example, vehicles, buses (e.g. Marjovi *et al*., 2015), trains, ships or ferries (e.g. Kelly‐Gerreyn *et al*., 2007) to provide a better and possibly more relevant coverage of the distributions of harmful compounds in the environment. Passive samplers and floating robots (Dong *et al*., 2014) already provide a wealth of data on basic physico‐chemical parameters (e.g. temperature, salinity), but might be combined with long‐lived biosensors targeting relevant toxic chemicals (Lohmann and Muir, 2010). Specific robots are being designed and constructed that can hold suits of miniaturized (bio)sensors, to be send on sampling missions in the environment and perhaps even capable to autonomously track sources of pollution (e.g. Fig. 3B and C). Data from (bio)sensor suites on static networks (e.g. buoys) as well as moving stations could become more widely accessible through Internet resources. Provided that some data pretreatment is performed this can help raise awareness to environmental quality issues and permit faster intervention. Finally, miniturized and cheaper biosensors will inevitably become integrated in small hand‐held devices available to the public. This will enable lay‐people to measure key biomedical and environmental parameters. Again, provided that proper data treatment and interpretation is given by such devices, this might help people in countries where government‐based monitoring is unreliable, to improve their awareness of environmental contamination issues.

## What is needed?

Despite successes and proof‐of‐concepts, the wider implementation of environmental biosensors is still a long way ahead. Few current biosensors are sufficiently robust and easy to allow wider application, and more practical experience is needed to improve such robustness. More comparative studies are needed to relate biosensor responses to chemical analyses (Siegfried *et al*., 2012) or to general ecotoxicity parameters, to make sense of the data and to decide which parameters are decisive (Jos *et al*., 2005; Vermeirssen *et al*., 2010; Brussaard *et al*., 2016). The advantage of biosensors is their potential direct deployment in the environment but their lack of sensitivity (Fig. 1) in direct environmental samples with often pM‐nM compound concentrations remains problematic. Therefore, more efforts are needed to develop automated sample pre‐concentration or purification methods that are compatible with downstream bioassays, e.g. by avoiding harmful organic solvents.

Despite covering a set of relevant toxic chemical compounds, there are many target molecules that cannot currently be effectively measured by biosensors, such as pharmaceuticals. More efforts are thus needed to engineer new sensing components in cell‐based or *in vitro* assays (e.g. antibodies, aptamers, protein design, regulatory proteins). It would also be important to accelerate the response time of cell‐based assays, in particular for hand‐held or robot applications that need a good quality readout after a few minutes. This may be difficult to achieve using classical reporter systems, but promising new ideas are circulating, such as biosensors based on changes in subcellular signal localization (Aymoz *et al*., 2016), on olfactory receptors (Lim *et al*., 2015) or chemotactic signalling (Sourjik *et al*., 2007). Miniaturization is a key for future bioassay applications, but to design and produce robust and simple devices that combine sample pretreatment, fluidics and bioassays is extremely challenging, and requires extensive collaboration between biologists, chemists and engineers. Finally, it will be crucial to convince legislators that under inclusion of proper synthetic engineering principles (e.g. no virulence genes, no antibiotic resistances, possible killing systems), it is timely to release restrictions on the lowest biosafety levels and admit that the risks in case of accidental liberation of biosensor cells are small in comparison to the gain of improved environmental quality monitoring.

## Conflict of interest

The authors declare no conflict of interest.
